# Gut Microbiota-Mediated Elevated Production of Secondary Bile Acids in Chronic Unpredictable Mild Stress

**DOI:** 10.3389/fphar.2022.837543

**Published:** 2022-03-07

**Authors:** Yuchen Qu, Cunjin Su, Qinhong Zhao, Aiming Shi, Fenglun Zhao, Liuxing Tang, Delai Xu, Zheng Xiang, Yang Wang, Yueyuan Wang, Jie Pan, Yunli Yu

**Affiliations:** ^1^ Department of Pharmacy, The Second Affiliated Hospital of Soochow University, Suzhou, China; ^2^ College of Pharmaceutical Science, Soochow University, Suzhou, China

**Keywords:** CUMS, bile acid, gut microbiota, depression, *Ruminococcaceae*

## Abstract

A growing body of evidence suggests that gut microbiota could participate in the progression of depression *via* the microbiota–gut–brain axis. However, the detailed microbial metabolic profile changes in the progression of depression is still not fully elucidated. In this study, a liquid chromatography coupled to mass spectrometry-based untargeted serum high-throughput metabolomics method was first performed to screen for potential biomarkers in a depressive-like state in a chronic unpredictable mild stress (CUMS)-induced mouse model. Our results identified that the bile acid and energy metabolism pathways were significantly affected in CUMS progression. The detailed bile acid profiles were subsequently quantified in the serum, liver, and feces. The results showed that CUMS significantly promoted the deconjugation of conjugated bile acid and secondary bile acid biosynthesis. Furthermore, 16S rRNA gene sequencing revealed that the increased secondary bile acid levels in the feces positively correlated with *Ruminococcaceae_UCG-010*, *Ruminococcus*, and *Clostridia_UCG-014* abundance. Taken together, our study suggested that changes in family *Ruminococcaceae* abundance following chronic stress increased biosynthesis of deoxycholic acid (DCA), a unconjugated secondary bile acid in the intestine. Aberrant activation of secondary bile acid biosynthesis pathway thereby increased the hydrophobicity of the bile acid pool, which might, in turn, promoted metabolic disturbances and disease progression in CUMS mice.

## Introduction

According to the World Health Organization, an estimated 3.8% of the global population has been affected by depression and the number is still increasing worldwide (World Health Organization, 2021). Modern psychology- and biology-related concepts revealed that depression is not only a common psychological disorder, but also a physical disease complex involving the imbalance of neurotransmitters, injury of neurogenesis, decline of neuroplasticity, and abnormality of neuronal circuitry (Chaudhury et al., 2015; Liu et al., 2017). Recently, with the development of gut microbiota research, a growing body of evidence indicates that the microbiota–gut–brain axis plays an essential role in regulating human behavior and brain function (Foster and McVey Neufeld, 2013; Liang et al., 2018).

An important function of the gut microbiota is participating in bile acid metabolism. Bile acids are the major constituents of the human bile synthesized from cholesterol by perivenous hepatocytes, playing an important role in dietary fat digestion and absorption (Hofmann., 1999). Most bile acids undergo enterohepatic circulation and microbial biotransformation in the intestinal tract (Chiang and Ferrell, 2018). Cholic and chenodeoxycholic acid are the two primary bile acids synthesized in the liver by a series of enzymatic reactions (Russell., 2003), conjugated with either glycine or taurine, and stored in the gallbladder (He et al., 2003). Bile acids are then secreted into the gastrointestinal tract, where they are subsequently deconjugated, dehydroxylated, and oxidized in the intestinal lumen by gut microbes to generate hydrophobic secondary bile acids: deoxycholic and lithocholic acid (Ridlon et al., 2014).

Recent studies revealed that bile acids might serve as intermediate messengers between the gut and the brain (Monteiro-Cardoso et al., 2021), while the relationship between bile acids and depression have rarely been investigated. In this study, we constructed a chronic unpredictable mild stress (CUMS) model to mimic depression-like symptoms in mice. We sought to explore potential gut microbiota-associated metabolites and the relationship between bile acid metabolic profiles and gut microbiota altered in CUMS progression.

## Materials and Methods

### Chemicals and Reagents

Cholic acid (CA), chenodeoxycholic acid (CDCA), ursodeoxycholic acid (UDCA), deoxycholic acid (DCA), lithocholic acid (LCA), taurocholic acid (TCA), taurochenodeoxycholic acid (TCDCA), tauroursodeoxycholic acid (TUDCA), taurodeoxycholic acid (TDCA), taurolithocholic acid (TLCA), glycocholic acid (GCA), glycochenodeoxycholic acid (GCDCA), glycoursodeoxycholic acid (GUDCA), glycodeoxycholic acid (GDCA), and lithocholic acid-2,2,3,4,4-d5 (internal standard) were all purchased from Sigma-Aldrich (St. Louis, MO, USA). Glycolithocholic acid (GLCA) was purchased from J&K Scientific Ltd. (Shanghai, China). HPLC-grade ammonium formate (≥99%), ammonium acetate, methanol, and acetonitrile were purchased from Merck KGaA (Darmstadt, Germany). HPLC-grade formic acid (99%) was purchased from Anaqua Chemicals Supply (Wilmington, USA).

### Animals and CUMS Experiment

Seven-week-old male ICR mice were purchased from SLAC Laboratory Animal Co., Ltd. (Shanghai, China). After their arrival, mice were single-caged and divided into the normal control group and the CUMS model group of 12 animals each randomly based on their body weight and sucrose preference test results. Mice were acclimated for 7 days in a temperature- (23–26°C) and humidity-controlled (40–60%) room under a 12-h light/dark cycle (lights on 07:00–19:00) with free access to food and water. During the modeling period, mice were weighed biweekly.

CUMS progression contained a total of 8 different stimulations including: 1) food deprivation for 24 h, 2) water deprivation for 24 h, 3) damp sawdust for 24 h, 4) tail pinching for 2 min, 5) restraint for 1 h, 6) cage tilting at 45° for 24 h, 7) cold swimming for 10 min, and 8) day and night reversal for 24 h. Two or three types of stimulations were delivered daily and randomly to the mice in the model groups for 56 days.

### Behavioral Tests and Sample Collection

Depression-related behavioral tests including the sucrose preference test (SPT), forced swim test (FST), and tail suspension test (TST) were performed during the experimental period.

For the SPT, all mice were habituated to 1% sucrose solution during the adaptation cycles. After the adaptation progression, mice were deprived of water and food for 12 h and were provided with free access to two tubes containing 20 ml of sucrose solution (1% w/v) and water respectively for 5 h. The sucrose preference rate was calculated subsequently using the following formula: sucrose preference = volume of sucrose consumed/total volume (water and sucrose) consumed × 100%. We performed the SPT on day 57 to evaluate the modeling effect.

We conducted the forced swim and tail suspension tests on days 58 and 59, respectively. During the forced swim test, the mice were individually placed into glass cylinders (height of 40 cm, diameter of 18 cm) containing 25°C water at a depth of 15 cm for 10 min. Immobility time was measured of last 4 min was recorded to estimate the symptom of depression. During the tail suspension test, mice were individually suspended by their tails for 6 min using a small piece of tape on the shelf, placed at the height of 60 cm above the floor. The duration of immobility during the final 4 min was recorded to measure depressive status.

On day 60, serum and feces were collected after 12 h of fasting. Livers and intestinal contents were removed immediately after the mice had been sacrificed. All samples were stored in a freezer at −80°C for further processing.

### Untargeted Metabolomic Analysis

Serum samples were thawed on ice and 400 µl of methanol was subsequently added into 100 µl of serum sample in an EP tube. The mixture was vortexed for 1 min and centrifuged at 15,000 g for 10 min at 4°C. The supernatant was then transferred into another EP tube and evaporated to dry with an Eppendorf Vacufuge Concentrator 5305. The residue was resuspended in 150 ul of 80% methanol and filtered through a 0.22-µm nylon syringe filter. For all samples, equal volumes of solutions were mixed into quality controlled samples to evaluate instrument analysis stability and repeatability.

The separation of the target compounds was performed on a Waters ACQUITY UPLC HSS T3 (2.1 mm × 150 mm, 1.8 µm) liquid chromatography column at 40°C with a ACQUITY UPLC CSH C18 VanGuard Pre-column (2.1 mm × 5 mm, 1.7 µm) using a Dionex Ultimate 3000 UPLC system. The mobile phase contained 0.1% aqueous formic acid and 0.1% formic acid in acetonitrile in positive ion mode and 5 mM ammonium formate aqueous buffer and acetonitrile in negative ion mode. The mobile phase flow rate was 0.25 ml/min and the injection volume was 5 µL both in the positive and negative ion modes. Supplementary Table S1 presents the detailed gradient elution conditions. The Q Exactive Orbitrap mass spectrometer (Thermo Fisher Scientific, USA) equipped with an ESI interface was applied for mass spectrometry analysis. The optimal parameters were as follows: sheath gas flow rate, 30 arb; aux gas flow rate, 10 arb; capillary temperature, 325°C; scan range: 81–1000 Da; stepped normalized collision energy, 30 in NCE mode; spray voltage, 3.5 kV (positive)/−2.5 kV (negative). All MS spectra were acquired and analyzed using the Xcalibur 4.0 software (Thermo Fisher Scientific).

### Data Processing and Metabolite Identification

Metabolomics analysis was carried out by BioNovoGene (Suzhou, China). After the raw data files were converted into an mzXML format by the ProteoWizard software (v3.0.8789), the freely available XCMS software was used to perform peak identification, filtration, alignment, and integration. The three-dimensional data matrix, including retention time, mass to charge ratio, and intensity, was converted into a table for further process analysis. In order to compare the data of different magnitudes, the peak area of the data was batch-normalized before multivariate statistical analysis. The data were then uploaded into SIMCA-P 13.0 to perform principal component analysis (PCA) and orthogonal partial least squares discriminant analysis (OPLS-DA). Autoscaling was used in all the models to achieve more scientific, reliable, and intuitive results. The variable importance in the project values (VIP) obtained from the OPLS-DA model and *p*-value from Student’s t-test were used to select the potential metabolites in the study. Metabolites with VIP >1 and *p*-value < 0.05 were considered statistically significant. These potential metabolites were subsequently subjected to pathway analysis performed through MetaboAnalyst 5.0 (https://www.metaboanalyst.ca/).

### Bile Acid Quantification in the Serum, Feces, and Liver

The bile acid profiles in the serum, feces, and liver were quantified using our previously validated UPLC-Q/Orbitrap-HRMS methods. Briefly, the bile acids in the feces and liver were extracted with 5 vol of deionized water by Qiagen TissueLyserII. For the feces samples, 200 uL of acetonitrile and 10 uL of 14% ammonia solution were added into 100 uL of fecal suspensions spiked with internal standard. For the serum and liver homogenates, 400 uL of acetonitrile was added into 100 uL of sample spiked with internal standard. The mixture was vortexed for 1 min and centrifuged at 20,000 g for 10 min at 4°C. The supernatant was then transferred into another EP tube and evaporated to dry with a vacuum centrifugal concentrator. The residue was resuspended in 100 uL of 80% methanol and filtered through a 0.22-µm nylon syringe filter. All blank matrix used for the calibration standard configurations and quality control samples were prepared using the activated carbon adsorption method.

The separation of the target compounds was performed on the same instrument and column as described above. The mobile phase flow rate and injection volume were 0.2 ml/min and 5 μL, respectively, with 10 mM ammonium formate aqueous buffer (A) and acetonitrile (B). The optimized gradient elution (0–7 min, 35–60% B; 7–8.5 min, 60–95% B; 8.5–12 min, 95% B; 12–12.3 min, 95%–35% B; 12.3–16 min, 35% B) was performed to separate the different bile acid components. Acquisition was performed in negative selective ion monitoring mode. All MS spectra were acquired and analyzed using the Xcalibur 4.0 software.

### 16S rRNA Sequencing Analysis

Total genomic DNA from the intestinal contents (5 samples from each of the control and model group) was extracted using the HiPure Stool DNA Kit (Megan, Guangzhou, China) according to the manufacturer’s protocols. The DNA concentration was measured using the Equalbit dsDNA HS Assay Kit (Novizan, Nanjing China). The NGS library preparation and Illumina sequencing was performed by GENEWIZ, Inc. (Suzhou, China). Approximately 20–30 ng of DNA was used to generate amplicons. The V3 and V4 hypervariable microbial 16S rDNA regions were amplified by PCR using a panel of proprietary primers designed by GENEWIZ. A linker with an index was then added to the end of the PCR product of 16S rDNA by PCR for NGS sequencing. The obtained sequencing library was subsequently purified with magnetic beads, followed by library quality control checks using a microplate reader and agarose gel electrophoresis. The library was then quantified to 10 nM and PE250/FE300 paired-end sequencing was performed using an Illumina MiSeq instrument (Illumina, San Diego, CA, USA).

Next, the forward and reverse reads were joined in pairs, followed by filtering the sequences containing N in the splicing results and retaining the sequences with a length beyond 200 bp. The obtained longer sequences were used to perform sequence clustering using VSEARCH (1.9.6) (sequence similarity was set to 97%) against the Silva_138 16SrRNA database (http://www.arb-silva.de/). The Ribosomal Database Program classifier was used to assign taxonomic categories to predict the community composition at the genus levels. Sequence data associated with this project have been deposited in the NCBI database (Accession Number: PRJNA796629).

### Statistical Analysis

All statistical analyses were performed using the GraphPad Prism 9.0 software. A two-tailed t-test was performed to compare between the groups and statistically significant differences were labeled with one, two, three, or four asterisks corresponding to *p* < 0.05, *p* < 0.01, or *p* < 0.001, respectively. Correlations between the gut microbiotic abundance and bile acid profiles were estimated using Pearson’s correlation analysis.

## Results

### Body Weight Changes and Depression-Like Behavior Validation

**FIGURE 1:**
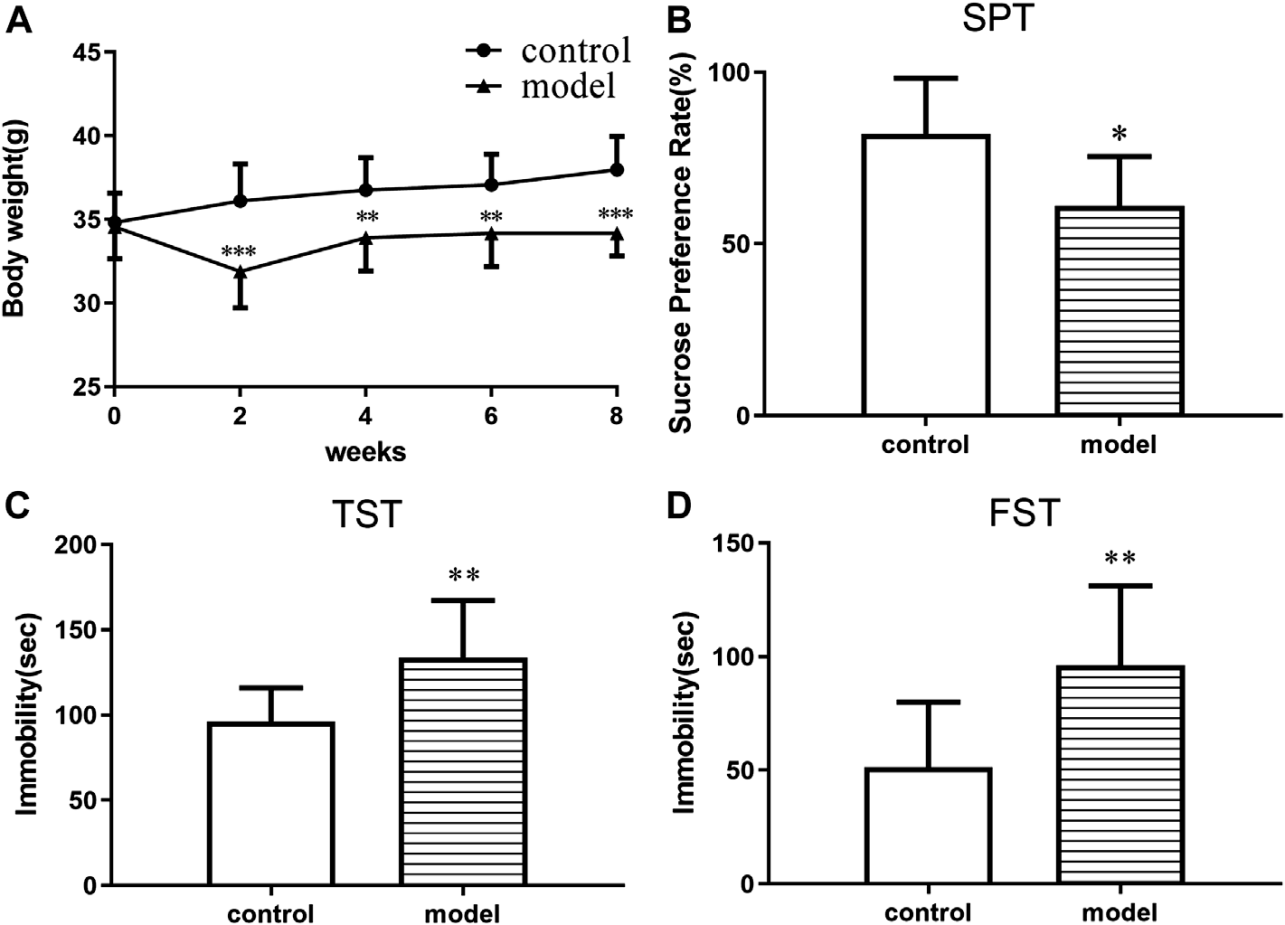
CUMS effects on body weight and depressive-like behaviors in ICR mice. **(A)** Body weight change. **(B)** Sucrose preference in the sucrose preference test. **(C)** Immobility time in the forced swimming test. **(D)** Immobility time in the tail suspension test. *p < 0.05. **p < 0.01. ***p < 0.001. Error bar, SD.

The body weight of the animals was measured before and during the treatment period. The mice in the model group gained less weight than control group at the end of the CUMS progression (Figures 1A–D). In addition, significant CUMS effects were present in the case of the sucrose consumption in SPT, immobility time in both FST and TST compared with the control group. The results demonstrated that a CUMS mouse model was successfully created.

### Differential Metabolite Screening in Untargeted Metabolomic Analysis

**FIGURE 2:**
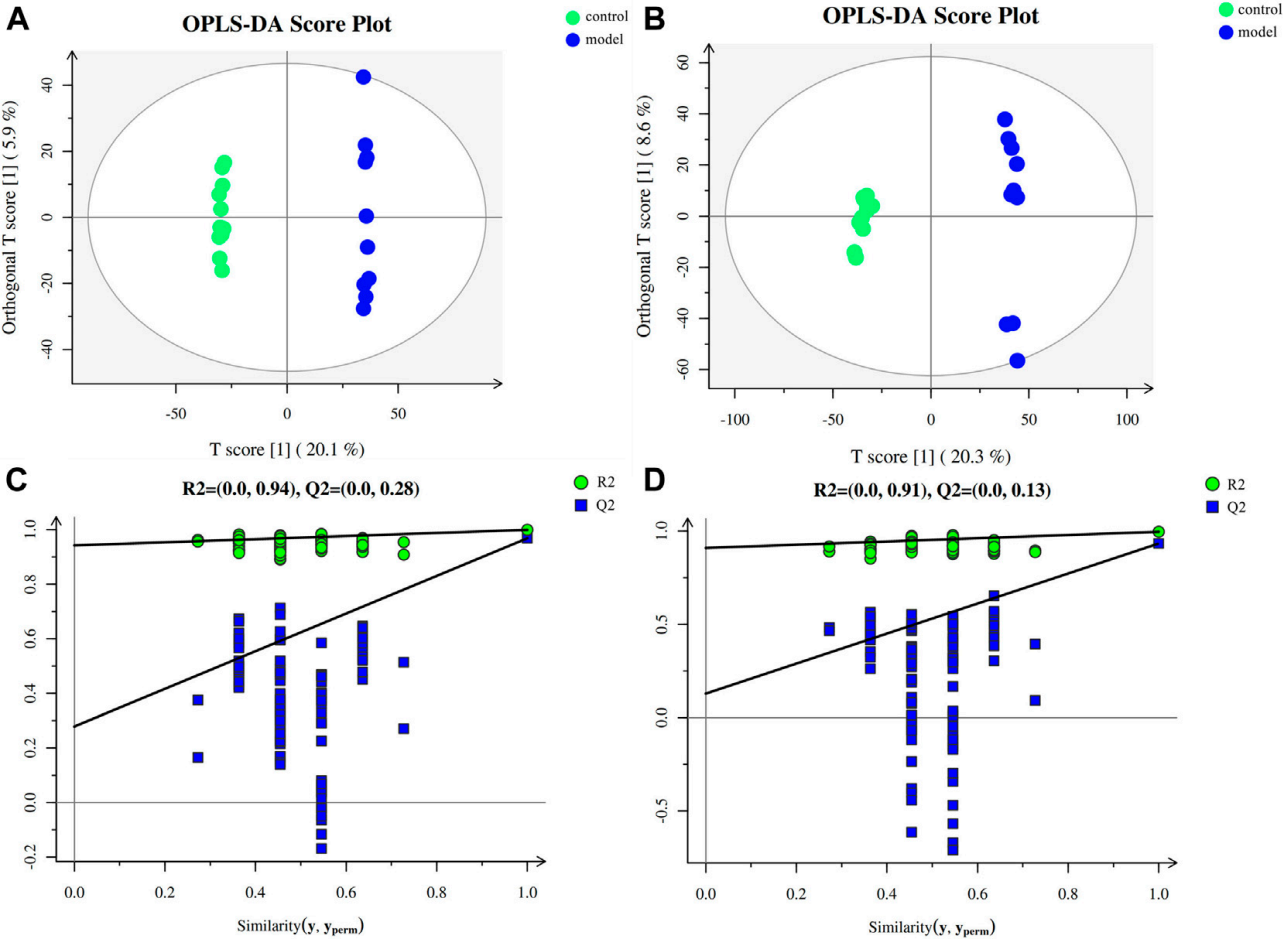
Multivariate data analysis and permutation test. **(A)** OPLS-DA score map for positive ion mode data. **(B)** OPLS-DA score map for negative ion mode data. **(C)** OPLS-DA permutation test for the positive ion mode data. **(D)** OPLS-DA permutation test for the negative ion mode data.

To investigate the impact of chronic stress upon the metabolomic profiling, untargeted metabolomic analysis was performed to analyze the metabolite composition in the serum of mice. The obvious separation trend from the PCA (Supplementary Figure S1) and the OPLS-DA (Figures 2A,B) score plot indicated metabolic differences between the groups. Our OPLS-DA permutation test showed that the model we established was not over-fitting (Figures 2C,D).

In order to screen out potential metabolites, we used the VIP value of the OPLS-DA model beyond 1.0 and the *p*-value of the two-tailed unpaired Student’s t-test results less than 0.05 as a threshold to distinguish the metabolites from the model and control groups. A total of 74 metabolites were significantly changed during the CUMS progression. Table 1 shows the detailed information of these metabolites. The affected pathways mainly involved amino acid, sugar, nucleotide metabolism, unsaturated fatty acid biosynthesis and metabolism, vitamin synthesis and absorption, and bile acid metabolism. Supplementary Figure S2A and Supplementary Table S2 show the bubble chart of the KEGG pathway analysis and the detailed information of the pathway analysis, respectively. We found that the two main primary bile acid (chenodeoxycholic acid and taurocholic acid) levels were significantly altered in the model group, indicating abnormalities in bile acid synthesis or metabolism.

**TABLE 1:** Identification of different metabolites.

Metabolites	KEGG ID	Model/control	Metabolites	KEGG ID	Model/control
S-Adenosylhomocysteine	C00021	↓	N-Acetylserotonin	C00978	↑
Pyruvic acid	C00022	↓	N-Acetyl-l-aspartic acid	C01042	↑
l-Glutamic acid	C00025	↑	4-Hydroxyphenylpyruvic acid	C01179	↓
Oxoglutaric acid	C00026	↑	Anserine	C01262	↑
l-Aspartic acid	C00049	↑	Linoleic acid	C01595	↑
l-Arginine	C00062	↑	Kynurenic acid	C01717	↑
l-Serine	C00065	↑	Pyroglutamic acid	C01879	↑
d-Fructose	C00095	↑	5-Methylcytosine	C02376	↑
2-Ketobutyric acid	C00109	↓	Xanthurenic acid	C02470	↑
Fumaric acid	C00122	↓	Chenodeoxycholic acid	C02528	↑
Adenine	C00147	↓	Ureidopropionic acid	C02642	↑
l-Proline	C00148	↑	N-Formyl-l-methionine	C03145	↓
5-Methylthioadenosine	C00170	↓	3-Hydroxykynurenine	C03227	↑
l-Lactic acid	C00186	↑	2-Oxoarginine	C03771	↓
3-Phosphoglyceric acid	C00197	↑	2-Dehydro-3-deoxy-l-rhamnonate	C03979	↑
Thymidine	C00214	↓	d-Octopine	C04137	↑
Butyric acid	C00246	↑	13-l-Hydroperoxylinoleic acid	C04717	↑
l-Sorbose	C00247	↓	Taurocholic acid	C05122	↓
Nicotinic acid	C00253	↓	Phenylethylamine	C05332	↑
Riboflavin	C00255	↑	beta-d-Fructose 6-phosphate	C05345	↑
Gluconic acid	C00257	↑	5(S)-HpETE	C05356	↓
Uridine	C00299	↓	Ergothioneine	C05570	↓
Retinal	C00376	↓	3,4-Dihydroxymandelic acid	C05580	↑
Carnosine	C00386	↑	Metanephrine	C05588	↑
*cis*-Aconitic acid	C00417	↑	5-Hydroxyindoleacetic acid	C05635	↑
Prostaglandin H2	C00427	↓	Prostaglandin G2	C05956	↓
Saccharopine	C00449	↓	Prostaglandin J2	C05957	↓
Nicotinamide ribotide	C00455	↓	6-Keto-prostaglandin F1a	C05961	↑
Retinol	C00473	↓	Salidroside	C06046	↓
Cytidine	C00475	↑	Skatole	C08313	↑
Glutaric acid	C00489	↓	13S-hydroxyoctadecadienoic acid	C14762	↑
l-Fucose	C00507	↓	12-KETE	C14807	↑
l-Arabinonate	C00545	↑	9(S)-HPODE	C14827	↑
5-Dehydro-4-deoxy-d-glucarate	C00679	↓	12,13-DHOME	C14829	↑
Betaine	C00719	↑	Stearidonic acid	C16300	↓
Glucaric acid	C00818	↓	Traumatic Acid	C16308	↑
Indole-3-acetic acid	C00954	↑	(2E,4Z,7Z,8E)-Colnelenic acid	C16320	↑

Different metabolites were identified from the OPLS-DA, model based on VIP > 1 and *p* < 0.05, ↑ indicates upregulated metabolites. ↓indicates downregulated metabolites.

### Effect of CUMS on Bile Acid Composition in the Serum

**FIGURE 3:**
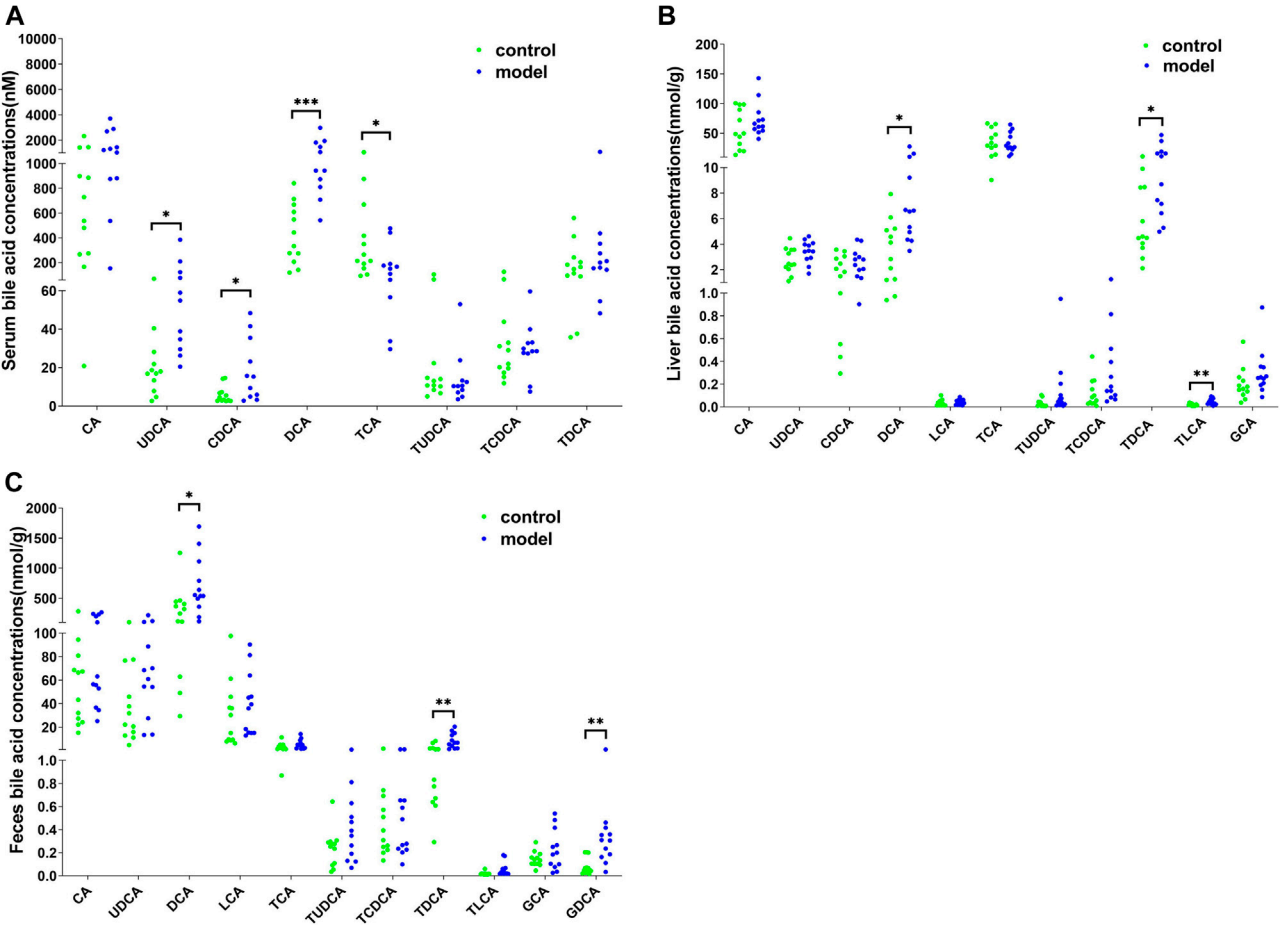
Detailed bile acid profiles in the **(A)** serum, **(B)** feces, and **(C)** liver. *p < 0.05. **p < 0.01. ***p < 0.001.

**FIGURE 4:**
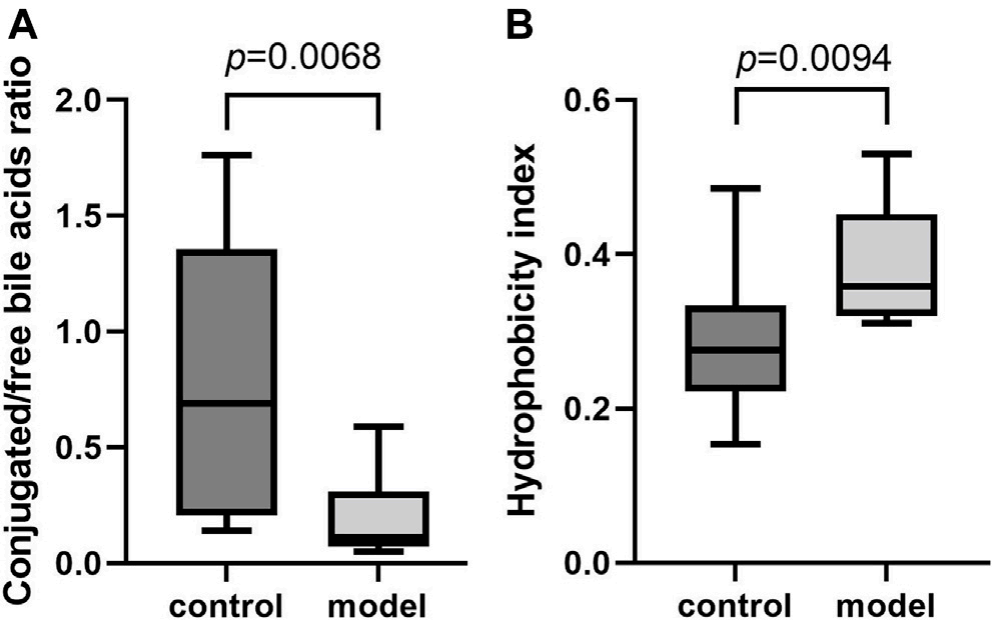
Comparisons of bile acid composition in the serum. **(A)** Boxplot for conjugated bile acid-to-free bile acid concentration ratio. **(B)** Boxplot for bile acid hydrophobicity index.

To further examine the bile acid metabolism disrupted by CUMS progression, we quantified the detailed bile acid profiles in the serum using our previously established method (Supplementary Figure S3 shows the chromatographic separation of the different components). CUMS significantly increased the level of three free bile acids, UDCA (345%↑, *p* = 0.0314), CDCA (220%↑, *p* = 0.0152), and DCA (197%↑, *p* = 0.0009), whereas it significantly reduced the level of taurine-conjugated primary bile acid TCA (56%↓, *p* = 0.0452) (Figure 3A). The taurine-conjugated-to-free bile acid ratios in the model group were significantly lower than those in the control group (Figure 4A). In addition, the hydrophobicity index (HI) of the circulating bile acid pool was calculated as described previously (Heuman, 1989). We observed that the HI in the model group was significantly higher than that in the control group (Figure 4B).

### Effect of CUMS on Bile Acid Composition in the Liver and Feces

Next, we quantified the bile acid profiles in the liver and feces to evaluate the effect of CUMS on bile acid biosynthesis and metabolism. CUMS significantly increased the secondary bile acid levels in the feces and liver (Figures 3B,C). In particular, the model group liver samples showed increased DCA (148%↑, *p* = 0.0198), TDCA (166%↑, *p* = 0.0222), and TLCA (137%↑, *p* = 0.0028) levels and the model group feces samples showed increased DCA (117%↑, *p* = 0.0343), TDCA (304%↑, *p* = 0.0034), and GDCA (306%↑, *p* = 0.0061) levels.

**FIGURE 5:**
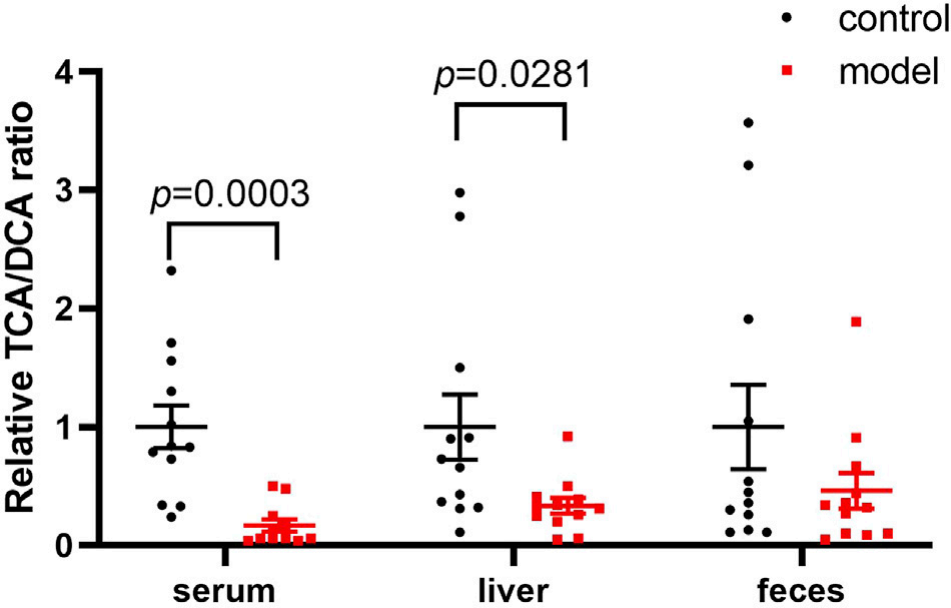
Comparisons of the relative TCA-to-DCA concentration ratio. Data represent ratio values normalized to percentage of model group and are shown as mean ± SEM.

Since DCA is the microbial metabolic product of TCA, we calculated the relative TCA-to-DCA ratios in the control and model groups to indirectly address the effects of the gut microbiota. The TCA/DCA ratio significantly decreased in the serum and liver of model group (Figure 5). Our results indicated that CUMS markedly promoted intestinal secondary bile acid formation.

### Association Between the Gut Microbiotic Abundance and Bile Acid Profiles

**FIGURE 6:**
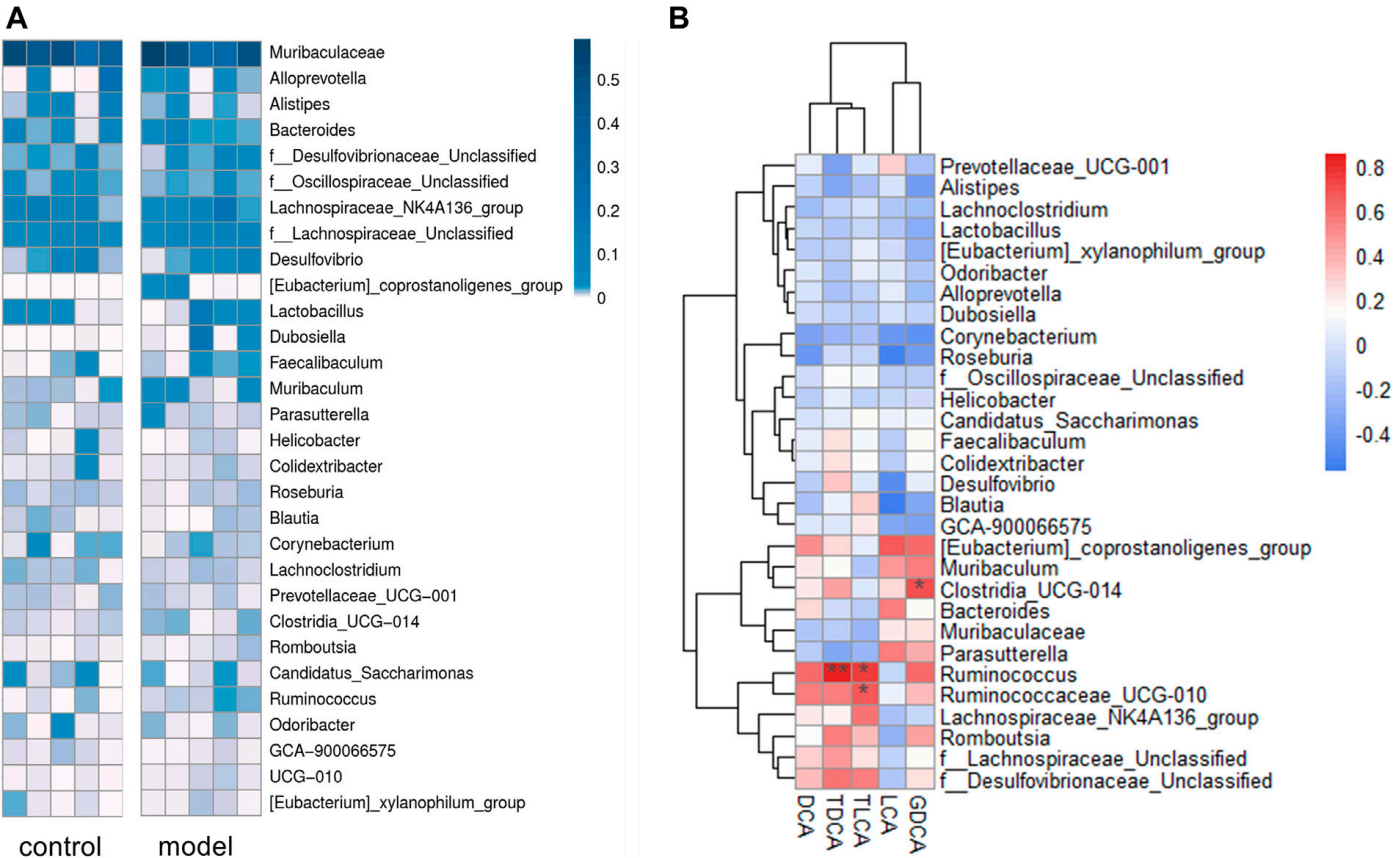
Sequencing data analysis at the generic level. **(A)** Heat map of the top 30 genera relative abundances. **(B)** Relationship between fecal secondary bile acid levels and the top 30 genera microbial relative abundances. The legends show the relative abundances and correlation values respectively. *p < 0.05. **p < 0.01.

We identified a total of 65 bacteria in the intestinal tract from the intestinal content samples at the genus level and summarized the heatmap of the relative abundance in the top 30 genera (Figure 6A). Pearson’s correlation analysis (Figure 6B) indicated that increased secondary bile acid levels in the feces significantly and positively correlated with three members of the phylum *Firmicutes*: *Ruminococcaceae_UCG-010*, *Ruminococcus*, and *Clostridia_UCG-014*. This result suggested that changes in the secondary bile acid formation might be associated with altered gut microbiota composition in the intestine.

## Discussion

In this study, we constructed a CUMS model to mimic depressive behavior in ICR mice with adverse stress in order to explore how depression could affect metabolism. These mice were weighed every 2 weeks and significant body weight gain reduction could be observed in model groups compared to the control. The SPT results showed that 8 weeks of CUMS significantly reduced sucrose solution consumption. The tail suspension and the forced swim tests are the most direct and effective methods to evaluate depressive behaviors in animals (Can et al., 2012; Slattery and Cryan, 2012). The immobility time of the model group during both the TST and FST significantly increased compared with that of the control. These behavioral results consistently supported that we successfully developed a CUMS model in ICR mice.

Metabolomics is an important component of systems biology, which can directly reflect the state of organisms (Fiehn, 2002). Our PCA and OPLS-DA score plots showed significant separation of the different groups, indicating that obvious metabolic differences occurred during CUMS progression. However, the pathway enrichment map revealed that the most impacted pathways mainly involved the energy metabolism, extensively studied in the field of depression. Upon further analysis of these metabolites, two primary bile acids were significantly altered in the model group, indicating that CUMS progression might cause bile acid metabolism disorder. In order to clarify how CUMS could affect bile acid metabolism, we quantified the detailed bile acid profiles in the serum, liver, and feces by UPLC-Q/Orbitrap-HRMS.

Bile acids are a group of amphipathic steroid molecules generated by hepatic and bacterial enzymes, playing an important role in regulating metabolism and immune response (Hofmann, 1999; Jia et al., 2018). Recent evidence suggests that bile acids might also play a role in mediating microbiota–gut–brain axis functions by interacting with their receptors in the brain (Monteiro-Cardoso et al., 2021). Specifically, altered bile acid profiles were associated with cognitive impairment in Alzheimer’s and Parkinson’s disease (MahmoudianDehkordi et al., 2019; Baloni et al., 2020; Li et al., 2021). Bile acid administration, particularly that of TUDCA and UDCA, contributed to neurologic symptom improvements in animal models of Alzheimer’s, Parkinson’s, and Huntington disease (Keene et al., 2002; Lo et al., 2013; Cuevas et al., 2020). However, bile acid metabolism in depression has rarely been described. There is an urgent need to elucidate how chronic stress affects bile acid profiles.

It is well-known that a variety of bile acid subtypes are present within the circulating bile acid pool (Yang et al., 2017). It has been difficult to appreciate the exact contribution of each bile acid to the whole body since each bile acid has the ability to bind and modulate the activity of transmembrane and nuclear receptors (Kundu et al., 2015). Different bile acid subtypes exhibit varying degrees of hydrophobicity, determined by factors such as state of ionization and hydroxyl group number, position, and orientation (Heuman, 1989). Circulating bile acid profile HIs quantitatively define the composite hydrophilic-hydrophobic balance of a mixture of bile acids (Heuman, 1989). Since multiple biological, physical, and chemical properties are related to the ability of compounds to bind to or dissolve in hydrophobic domains such as membrane, micelles, or certain receptor sites, HI can be used to evaluate how bile acid profile alterations impact body function (Haeusler et al., 2013). Therefore, we calculated the circulating bile acid pool HI in the serum and observed remarkably raised bile acid pool HI in the model group.

Apart from HI, the conjugated/free and primary/secondary form ratios are additional characteristics of host bile acid homeostasis. We observed that the conjugated-to-free form ratios in the serum significantly reduced in the model group. In addition, secondary bile acid levels, especially that of DCA, markedly increased in the model group. Since DCA is the metabolic product of TCA, we further compared the TCA/DCA ratio in control and model groups. Consequently, the TCA/DCA ratios in the serum and liver significantly decreased in the model group. These results indicated that the secondary bile acid biosynthesis pathway had been activated during CUMS progression, potentially explaining the increased HI in the serum.

Mitochondrial dysfunction and oxidative stress are supposed to be involved in the pathophysiology of depression (Bansal and Kuhad, 2016; Bhatt et al., 2020). Indeed, hydrophobic bile acid species show cytotoxicity due to their detergent action and oxidation effects (Perez and Briz, 2009), whereas hydrophilic bile acids, such as TUDCA mentioned above, exert strong cytoprotective effects by mitochondrial membrane stabilization (Castro et al., 2004). Previous studies demonstrated that multiple bile acids could penetrate the blood-brain barrier, although the involved mechanisms have not yet been fully understood (Mertens et al., 2017). Notably, free bile acids could diffuse across phospholipid bilayers and their brain concentrations correlate with their serum concentrations (Kamp et al., 1993; Higashi et al., 2017). Since chronic stress can disrupt brain homeostasis and increase the blood-brain barrier permeability (Lee et al., 2018), free bile acids might penetrate more easily the blood-brain barrier in the depressive state. In particular, increased DCA level can induce apoptosis and DNA damage (Washo-Stultz et al., 2002; Fu et al., 2019), potentially exacerbating neuroinflammation and oxidative stress contributing to the progression of depression pathology. In our analyses, with CUMS progression, the increased free bile acid levels in serum might have contributed to bile acid composition changes in the brain. This is of particular concern given that the increased DCA level might affect brain physiology.

In addition to direct effects on the central nervous system function, bile acids might also be involved in the metabolic disorders during CUMS progression by regulating receptor such as FXR (farnesoid X receptor) and TGR5 (Takeda G protein-coupled receptor 5). FXR mainly functions as a bile acid sensor in the bile acid regulation feedback and its most potent ligand is CDCA (Liu et al., 2020). Furthermore, current evidence suggests FXR also participates in bile acid-mediated energy metabolism. FXR deficient mice exhibited impaired glucose tolerance and reduced insulin sensitivity (Ma et al., 2006). FXR activation in the intestine promotes the release of fibroblast growth factor (FGF) 15/19, proved to serve as important regulators to improve glucose metabolism in the gut–brain axis by binding FGF receptors in the hypothalamus (Liu et al., 2018). TGR5 is a G protein-coupled bile acid receptor that mediates glucose homeostasis by producing glucagon-like peptide 1. TGR5 is mainly activated by secondary bile acids, including LCA, DCA, and TLCA *in vivo* (Liu et al., 2020). Our serum untargeted metabolomics results revealed multiple carbohydrate metabolism pathways were significantly enriched, which might be associated with the bile acid metabolism disorders in CUMS progression. Beyond that, the latest research indicated that bile acid receptors in the brain were also directly involved in the pathogenesis of depression. The protein and mRNA expressions of FXR in hippocampus were significantly increased in CUMS induced depressive rats (Chen et al., 2018), and FXR overexpression aggravated depression-like behaviors by inhibiting brain-derived neurotrophic factor signaling in the hippocampus (Hu et al., 2020). Multiple types of chronic stressors significantly reduced TGR5 expression in hippocampal CA3 pyramidal neurons of C57BL/6J mice, whereas genetic overexpression of TGR5 or intra-CA3 infusion of the TGR5 agonist was able to reverse depressive-like behaviors by CA3 pyramidal neurons activation (Wang et al., 2021). In our present study, hepatic FXR and TGR5 expressions were not significantly altered in CUMS progression (data not shown), indicating that CUMS might exert different effects on bile acid reporters in different tissues. Bile acid profile and FXR and TGR5 expression in the brain will be the focus of our further investigation to elucidate the regulation of the pathogenesis of depression.

It is known that gut microbiota plays an essential role in the development of depression (Sanada et al., 2020), recognized as a possible reason for depression causing bile acid metabolism disorder. The deconjugation of conjugated bile acids *in vivo* is mainly catalyzed by bile salt hydrolase, widely expressed by multiple common commensal genera, especially *Bacteroides*, *Lactobacillus,* and *Clostridium* (Song et al., 2019; Adhikari et al., 2020). Another microbial bile salt transformation *in vivo* is to form secondary bile acids from primary bile acids by 7α-dehydroxylation. Currently known bacteria expressing 7α-dehydroxylase are all of the *Ruminococcaceae* and *Lachnospiraceae* families (Stellwag and Hylemon, 1978; Takamine and Imamura, 1995). As shown in Supplementary Figure S4, two genera of family *Ruminococcaceae*, *Ruminococcaceae_UCG-010* and *Ruminococcus*, were significantly positively correlated with the secondary bile acid levels in feces, which might partly explain the increased secondary bile acids in model group.

Accumulating number of studies demonstrated that *Ruminococcaceae* might affect brain function and behavior. Tran et al. found that the abundance of *Ruminococcaceae* were correlated with the apolipoprotein E genotype in healthy participants (Tran et al., 2019). Depletion of *Ruminococcaceae* was proved to be closely associated with reduced cognitive functions Alzheimer’s disease (Vogt et al., 2017; D'Amato et al., 2020). At the genus level, the genus *Ruminococcus* is well known as butyric acid-producing bacteria which plays an important role in intestinal inflammation (Louis and Flint, 2017; Henke et al., 2019) (elevated butyric acid level was also observed in model group from the serum untargeted metabolomics data). Recent research revealed the importance of decreasing *Ruminococcus* for duloxetine to reduce depressive behavior (Lukić et al., 2019), indirectly indicating that *Ruminococcus* involved in the occurrence and progression of depression. Our current results further complement these previous study findings demonstrating the critical role of *Ruminococcaceae* by regulating bile acid metabolism in microbiota–gut–brain axis.

## Conclusion

Our findings provide a novel perspective to elucidate the microbiota–gut–brain crosstalk in depression. Chronic stress-induced gut microbiota modifications, especially changes in relative abundance of family *Ruminococcaceae*, contributed to increased biosynthesis of secondary bile acid DCA in the intestine. This gut microbiota-mediated bile acid metabolic imbalance subsequently increased the hydrophobicity of the bile acid pool, which might in turn promote the energy metabolism disorder and pathophysiological changes in CUMS progression.

## Data Availability

The datasets presented in this study can be found in online repositories. The names of the repository/repositories and accession number(s) can be found below: NCBI database (BioProject: PRJNA796629, BioSample:SAMN24905304).

## References

[B1] Adhikari A. A. Seegar T. C. M. Ficarro S. B. McCurry M. D. Ramachandran D. Yao L. (2020). Development of a Covalent Inhibitor of Gut Bacterial Bile Salt Hydrolases. Nat. Chem. Biol. 16 (3), 318–326. 10.1038/s41589-020-0467-3 32042200 PMC7036035

[B2] Baloni P. Funk C. C. Yan J. Yurkovich J. T. Kueider-Paisley A. Nho K. (2020). Metabolic Network Analysis Reveals Altered Bile Acid Synthesis and Metabolism in Alzheimer's Disease. Cell. Rep. Med. 1 (8), 100138. 10.1016/j.xcrm.2020.100138 33294859 PMC7691449

[B3] Bansal Y. Kuhad A. (2016). Mitochondrial Dysfunction in Depression. Curr. Neuropharmacol. 14 (6), 610–618. 10.2174/1570159x14666160229114755 26923778 PMC4981740

[B4] Bhatt S. Nagappa A. N. Patil C. R. (2020). Role of Oxidative Stress in Depression. Drug Discov. Today 25 (7), 1270–1276. 10.1016/j.drudis.2020.05.001 32404275

[B5] Can A. Dao D. T. Terrillion C. E. Piantadosi S. C. Bhat S. Gould T. D. (2012). The Tail Suspension Test. J. Vis. Exp. (59), e3769. 10.3791/3769 22315011 PMC3353516

[B6] Castro R. E. Solá S. Ramalho R. M. Steer C. J. Rodrigues C. M. (2004). The Bile Acid Tauroursodeoxycholic Acid Modulates Phosphorylation and Translocation of Bad via Phosphatidylinositol 3-kinase in Glutamate-Induced Apoptosis of Rat Cortical Neurons. J. Pharmacol. Exp. Ther. 311 (2), 845–852. 10.1124/jpet.104.070532 15190125

[B7] Chaudhury D. Liu H. Han M. H. (2015). Neuronal Correlates of Depression. Cell. Mol. Life Sci. 72, 4825–4848. 10.1007/s00018-015-2044-6 26542802 PMC4709015

[B8] Chen W. G. Zheng J. X. Xu X. Hu Y. M. Ma Y. M. (2018). Hippocampal FXR Plays a Role in the Pathogenesis of Depression: A Preliminary Study Based on Lentiviral Gene Modulation. Psychiatry Res. 264, 374–379. 10.1016/j.psychres.2018.04.025 29677620

[B9] Chiang J. Y. L. Ferrell J. M. (2018). Bile Acid Metabolism in Liver Pathobiology. Gene Expr. 18 (2), 71–87. 10.3727/105221618X15156018385515 29325602 PMC5954621

[B10] Cuevas E. Burks S. Raymick J. Robinson B. Gómez-Crisóstomo N. P. Escudero-Lourdes C. (2020). Tauroursodeoxycholic Acid (TUDCA) Is Neuroprotective in a Chronic Mouse Model of Parkinson's Disease. Nutr. Neurosci. 1, 1–18. 10.1080/1028415X.2020.1859729 33345721

[B11] D'Amato A. Di Cesare Mannelli L. Lucarini E. Man A. L. Le Gall G. Branca J. J. V. (2020). Faecal Microbiota Transplant from Aged Donor Mice Affects Spatial Learning and Memory via Modulating Hippocampal Synaptic Plasticity- and Neurotransmission-Related Proteins in Young Recipients. Microbiome 8 (1), 140. 10.1186/s40168-020-00914-w 33004079 PMC7532115

[B12] Fiehn O. (2002). Metabolomics--the Link between Genotypes and Phenotypes. Plant Mol. Biol. 48 (1-2), 155–171. 10.1007/978-94-010-0448-0_11 11860207

[B13] Foster J. A. McVey Neufeld K. A. (2013). Gut-brain axis: How the Microbiome Influences Anxiety and Depression. Trends. Neurosci. 36 (5), 305–312. 10.1016/j.tins.2013.01.005 23384445

[B14] Fu T. Coulter S. Yoshihara E. Oh T. G. Fang S. Cayabyab F. (2019). FXR Regulates Intestinal Cancer Stem Cell Proliferation. Cell 176 (5), 1098–1112e18. 10.1016/j.cell.2019.01.036 30794774 PMC6701863

[B15] Haeusler R. A. Astiarraga B. Camastra S. Accili D. Ferrannini E. (2013). Human Insulin Resistance Is Associated with Increased Plasma Levels of 12α-Hydroxylated Bile Acids. Diabetes 62 (12), 4184–4191. 10.2337/db13-0639 23884887 PMC3837033

[B16] He D. Barnes S. Falany C. N. (2003). Rat Liver Bile Acid CoA:amino Acid N-Acyltransferase: Expression, Characterization, and Peroxisomal Localization. J. Lipid. Res. 44 (12), 2242–2249. 10.1194/jlr.M300128-JLR200 12951368

[B17] Henke M. T. Kenny D. J. Cassilly C. D. Vlamakis H. Xavier R. J. Clardy J. (2019). Ruminococcus Gnavus, a Member of the Human Gut Microbiome Associated with Crohn's Disease, Produces an Inflammatory Polysaccharide. Proc. Natl. Acad. Sci. U. S. A. 116 (26), 12672–12677. 10.1073/pnas.1904099116 31182571 PMC6601261

[B18] Heuman D. M. (1989). Quantitative Estimation of the Hydrophilic-Hydrophobic Balance of Mixed Bile Salt Solutions. J. Lipid. Res. 30 (5), 719–730. 10.1016/s0022-2275(20)38331-0 2760545

[B19] Higashi T. Watanabe S. Tomaru K. Yamazaki W. Yoshizawa K. Ogawa S. (2017). Unconjugated Bile Acids in Rat Brain: Analytical Method Based on LC/ESI-MS/MS with Chemical Derivatization and Estimation of Their Origin by Comparison to Serum Levels. Steroids 125, 107–113. 10.1016/j.steroids.2017.07.001 28689738

[B20] Hofmann A. F. (1999). The Continuing Importance of Bile Acids in Liver and Intestinal Disease. Arch. Intern. Med. 159 (22), 2647–2658. 10.1001/archinte.159.22.2647 10597755

[B21] Hu W. Wu J. Ye T. Chen Z. Tao J. Tong L. (2020). Farnesoid X Receptor-Mediated Cytoplasmic Translocation of CRTC2 Disrupts CREB-BDNF Signaling in Hippocampal CA1 and Leads to the Development of Depression-like Behaviors in Mice. Int. J. Neuropsychopharmacol. 23 (10), 673–686. 10.1093/ijnp/pyaa039 32453814 PMC7727490

[B22] Jia W. Xie G. Jia W. (2018). Bile Acid-Microbiota Crosstalk in Gastrointestinal Inflammation and Carcinogenesis. Nat. Rev. Gastroenterol. Hepatol. 15 (2), 111–128. 10.1038/nrgastro.2017.119 29018272 PMC5899973

[B23] Kamp F. Hamilton J. A. Kamp F. Westerhoff H. V. Hamilton J. A. (1993). Movement of Fatty Acids, Fatty Acid Analogues, and Bile Acids across Phospholipid Bilayers. Biochemistry 32 (41), 11074–11086. 10.1021/bi00092a017 8218171

[B24] Keene C. D. Rodrigues C. M. Eich T. Chhabra M. S. Steer C. J. Low W. C. (2002). Tauroursodeoxycholic Acid, a Bile Acid, Is Neuroprotective in a Transgenic Animal Model of Huntington's Disease. Proc. Natl. Acad. Sci. U. S. A. 99 (16), 10671–10676. 10.1073/pnas.162362299 12149470 PMC125009

[B25] Kundu S. Kumar S. Bajaj A. (2015). Cross-talk between Bile Acids and Gastrointestinal Tract for Progression and Development of Cancer and its Therapeutic Implications. IUBMB. Life 67 (7), 514–523. 10.1002/iub.1399 26177921

[B26] Lee S. Kang B. M. Kim J. H. Min J. Kim H. S. Ryu H. (2018). Real-time *In Vivo* Two-Photon Imaging Study Reveals Decreased Cerebro-Vascular Volume and Increased Blood-Brain Barrier Permeability in Chronically Stressed Mice. Sci. Rep. 8 (1), 13064. 10.1038/s41598-018-30875-y 30166586 PMC6117335

[B27] Li P. Killinger B. A. Ensink E. Beddows I. Yilmaz A. Lubben N. (2021). Gut Microbiota Dysbiosis Is Associated with Elevated Bile Acids in Parkinson's Disease. Metabolites 11 (1), 29. 10.3390/metabo11010029 33406628 PMC7823437

[B28] Liang S. Wu X. Hu X. Wang T. Jin F. (2018). Recognizing Depression from the Microbiota⁻Gut⁻Brain Axis. Int. J. Mol. Sci. 19 (6), 1592. 10.3390/ijms19061592 PMC6032096 29843470

[B29] Liu B. Liu J. Wang M. Zhang Y. Li L. (2017). From Serotonin to Neuroplasticity: Evolvement of Theories for Major Depressive Disorder. Front. Cel. Neurosci. 11, 305. 10.3389/fncel.2017.00305 PMC5624993 29033793

[B30] Liu L. Liu Z. Li H. Cao Z. Li W. Song Z. (2020). Naturally Occurring TPE-CA Maintains Gut Microbiota and Bile Acids Homeostasis via FXR Signaling Modulation of the Liver-Gut Axis. Front. Pharmacol. 11, 12. 10.3389/fphar.2020.00012 32116693 PMC7015895

[B31] Liu S. Marcelin G. Blouet C. Jeong J. H. Jo Y. H. Schwartz G. J. (2018). A Gut-Brain axis Regulating Glucose Metabolism Mediated by Bile Acids and Competitive Fibroblast Growth Factor Actions at the Hypothalamus. Mol. Metab. 8, 37–50. 10.1016/j.molmet.2017.12.003 29290621 PMC5985052

[B32] Lo A. C. Callaerts-Vegh Z. Nunes A. F. Rodrigues C. M. D'Hooge R. (2013). Tauroursodeoxycholic Acid (TUDCA) Supplementation Prevents Cognitive Impairment and Amyloid Deposition in APP/PS1 Mice. Neurobiol. Dis. 50, 21–29. 10.1016/j.nbd.2012.09.003 22974733

[B33] Louis P. Flint H. J. (2017). Formation of Propionate and Butyrate by the Human Colonic Microbiota. Environ. Microbiol. 19 (1), 29–41. 10.1111/1462-2920.13589 27928878

[B34] Lukić I. Getselter D. Ziv O. Oron O. Reuveni E. Koren O. (2019). Antidepressants Affect Gut Microbiota and Ruminococcus Flavefaciens Is Able to Abolish Their Effects on Depressive-like Behavior. Transl. Psychiatry 9 (1), 133. 10.1038/s41398-019-0466-x 30967529 PMC6456569

[B35] Ma K. Saha P. K. Chan L. Moore D. D. (2006). Farnesoid X Receptor Is Essential for normal Glucose Homeostasis. J. Clin. Invest. 116 (4), 1102–1109. 10.1172/JCI25604 16557297 PMC1409738

[B36] MahmoudianDehkordi S. Arnold M. Nho K. Ahmad S. Jia W. Xie G. (2019). Altered Bile Acid Profile Associates with Cognitive Impairment in Alzheimer's Disease-An Emerging Role for Gut Microbiome. Alzheimers. Dement. 15 (1), 76–92. 10.1016/j.jalz.2018.07.217 30337151 PMC6487485

[B37] Mertens K. L. Kalsbeek A. Soeters M. R. Eggink H. M. (2017). Bile Acid Signaling Pathways from the Enterohepatic Circulation to the Central Nervous System. Front. Neurosci. 11, 617. 10.3389/fnins.2017.00617 29163019 PMC5681992

[B38] Monteiro-Cardoso V. F. Corlianò M. Singaraja R. R. (2021). Bile Acids: A Communication Channel in the Gut-Brain Axis. Neuromolecular. Med. 23 (1), 99–117. 10.1007/s12017-020-08625-z 33085065

[B39] Perez M. J. Briz O. (2009). Bile-acid-induced Cell Injury and protection. World J. Gastroenterol. 15 (14), 1677–1689. 10.3748/wjg.15.1677 19360911 PMC2668773

[B40] Ridlon J. M. Kang D. J. Hylemon P. B. Bajaj J. S. (2014). Bile Acids and the Gut Microbiome. Curr. Opin. Gastroenterol. 30 (3), 332–338. 10.1097/MOG.0000000000000057 24625896 PMC4215539

[B41] Russell D. W. (2003). The Enzymes, Regulation, and Genetics of Bile Acid Synthesis. Annu. Rev. Biochem. 72, 137–174. 10.1146/annurev.biochem.72.121801.161712 12543708

[B42] Sanada K. Nakajima S. Kurokawa S. Barceló-Soler A. Ikuse D. Hirata A. (2020). Gut Microbiota and Major Depressive Disorder: A Systematic Review and Meta-Analysis. J. Affect. Disord. 266, 1–13. 10.1016/j.jad.2020.01.102 32056863

[B43] Slattery D. A. Cryan J. F. (2012). Using the Rat Forced Swim Test to Assess Antidepressant-like Activity in Rodents. Nat. Protoc. 7 (6), 1009–1014. 10.1038/nprot.2012.044 22555240

[B44] Song Z. Cai Y. Lao X. Wang X. Lin X. Cui Y. (2019). Taxonomic Profiling and Populational Patterns of Bacterial Bile Salt Hydrolase (BSH) Genes Based on Worldwide Human Gut Microbiome. Microbiome 7 (1), 9. 10.1186/s40168-019-0628-3 30674356 PMC6345003

[B45] Stellwag E. J. Hylemon P. B. (1978). Characterization of 7-Alpha-Dehydroxylase in Clostridium Leptum. Am. J. Clin. Nutr. 31 (10 Suppl. l), S243–S247. 10.1093/ajcn/31.10.S243 707382

[B46] Takamine F. Imamura T. (1995). Isolation and Characterization of Bile Acid 7-dehydroxylating Bacteria from Human Feces. Microbiol. Immunol. 39 (1), 11–18. 10.1111/j.1348-0421.1995.tb02162.x 7783673

[B47] Tran T. T. T. Corsini S. Kellingray L. Hegarty C. Le Gall G. Narbad A. (2019). APOE Genotype Influences the Gut Microbiome Structure and Function in Humans and Mice: Relevance for Alzheimer's Disease Pathophysiology. FASEB j. 33, 8221–8231. 10.1096/fj.201900071R 30958695 PMC6593891

[B48] Vogt N. M. Kerby R. L. Dill-McFarland K. A. Harding S. J. Merluzzi A. P. Johnson S. C. (2017). Gut Microbiome Alterations in Alzheimer's Disease. Sci. Rep. 7 (1), 13537. 10.1038/s41598-017-13601-y 29051531 PMC5648830

[B49] Wang H. Tan Y. Z. Mu R. H. Tang S. S. Liu X. Xing S. Y. (2021). Takeda G Protein-Coupled Receptor 5 Modulates Depression-like Behaviors via Hippocampal CA3 Pyramidal Neurons Afferent to Dorsolateral Septum. Biol. Psychiatry 89 (11), 1084–1095. 10.1016/j.biopsych.2020.11.018 33536132

[B50] Washo-Stultz D. Crowley-Weber C. L. Dvorakova K. Bernstein C. Bernstein H. Kunke K. (2002). Role of Mitochondrial Complexes I and II, Reactive Oxygen Species and Arachidonic Acid Metabolism in Deoxycholate-Induced Apoptosis. Cancer Lett. 177 (2), 129–144. 10.1016/s0304-3835(01)00786-8 11825660

[B51] World Health Organization (WHO) (2021). Depression. Available online at: https://www.who.int/en/news-room/fact-sheets/detail/depression .

[B52] Yang T. Shu T. Liu G. Mei H. Zhu X. Huang X. (2017). Quantitative Profiling of 19 Bile Acids in Rat Plasma, Liver, Bile and Different Intestinal Section Contents to Investigate Bile Acid Homeostasis and the Application of Temporal Variation of Endogenous Bile Acids. J. Steroid Biochem. Mol. Biol. 172, 69–78. 10.1016/j.jsbmb.2017.05.015 28583875

