# Supplementary material for: Gut Microbiota-Mediated Elevated Production of Secondary Bile Acids in Chronic Unpredictable Mild Stress
Source: Front Pharmacol. 2022 Mar 7;13:837543. doi: 10.3389/fphar.2022.837543 (PMC8936594; doi:10.3389/fphar.2022.837543)
Supplement: Supplementary file 1 [file DataSheet1.docx]

Supplementary Material


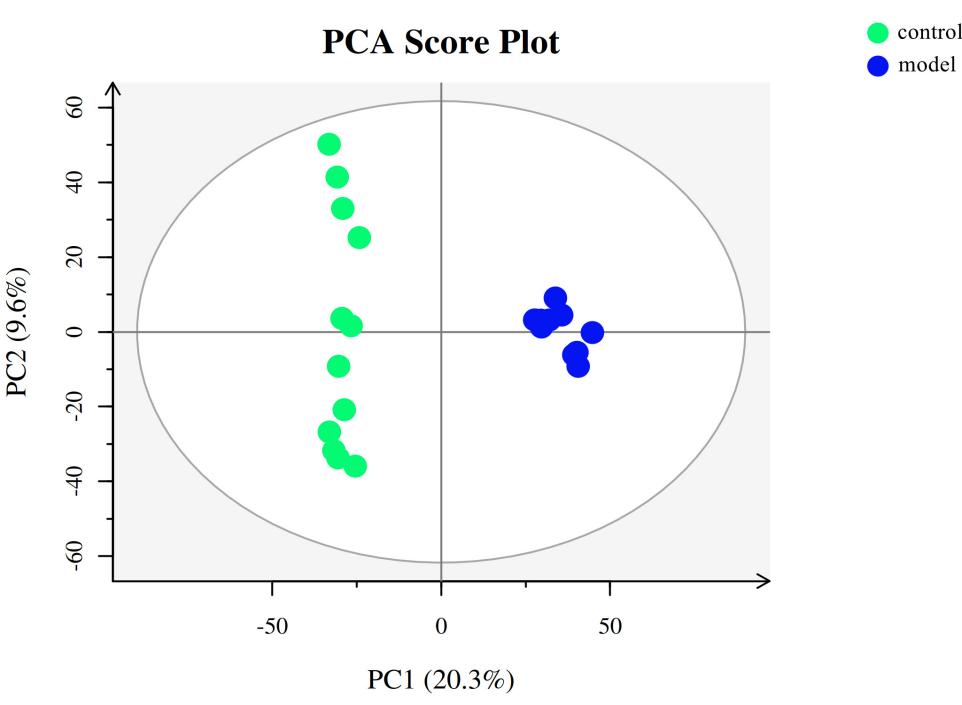

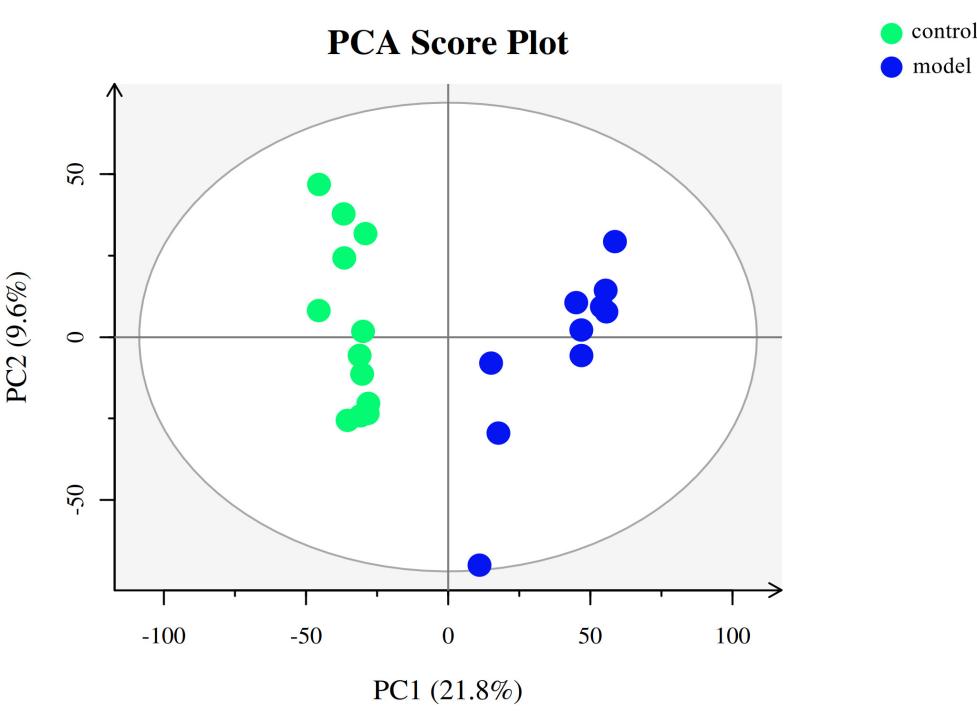


A

B

**Supplementary Figure S1.** PCA score plot of (A) positive ion mode data and (B) negative ion mode data.


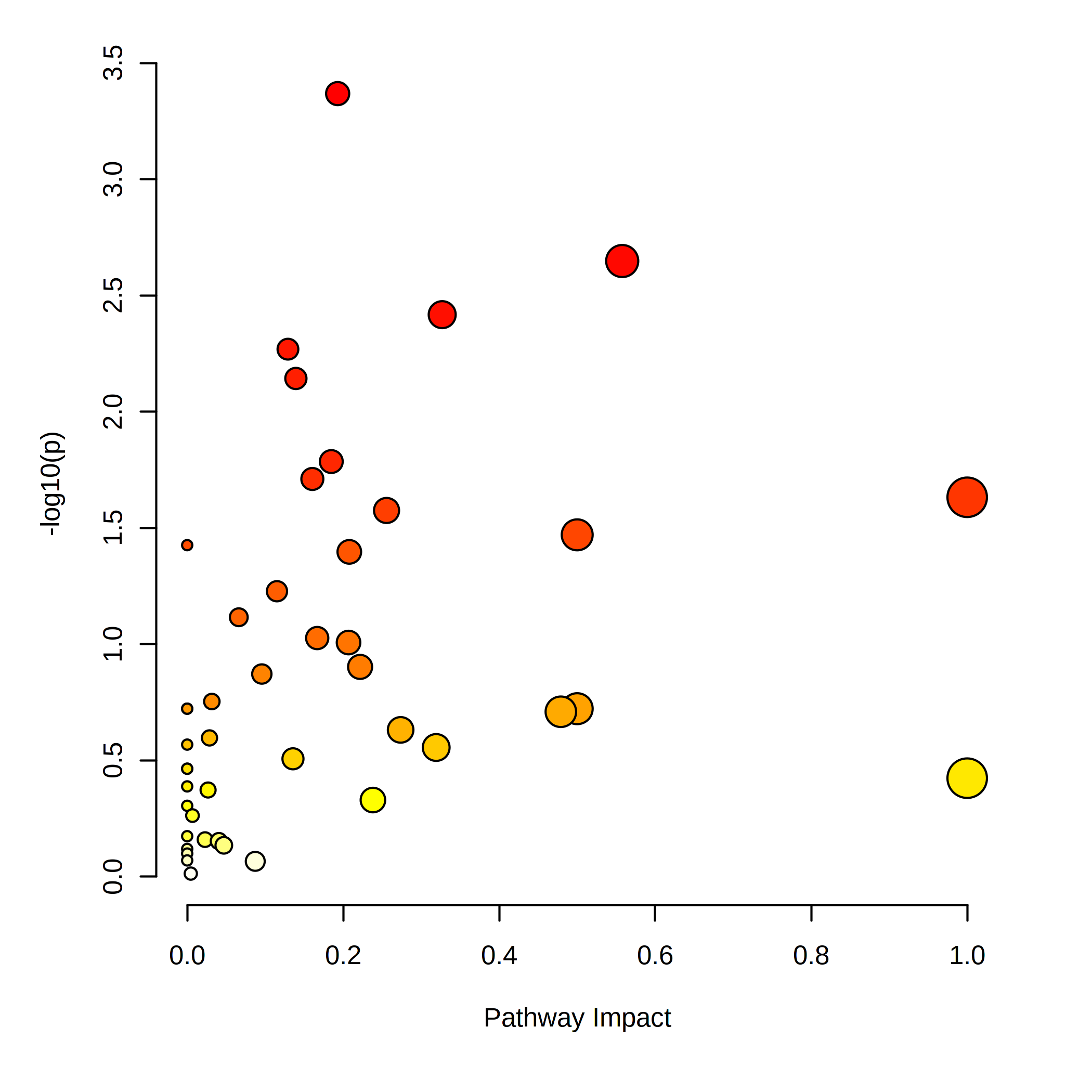

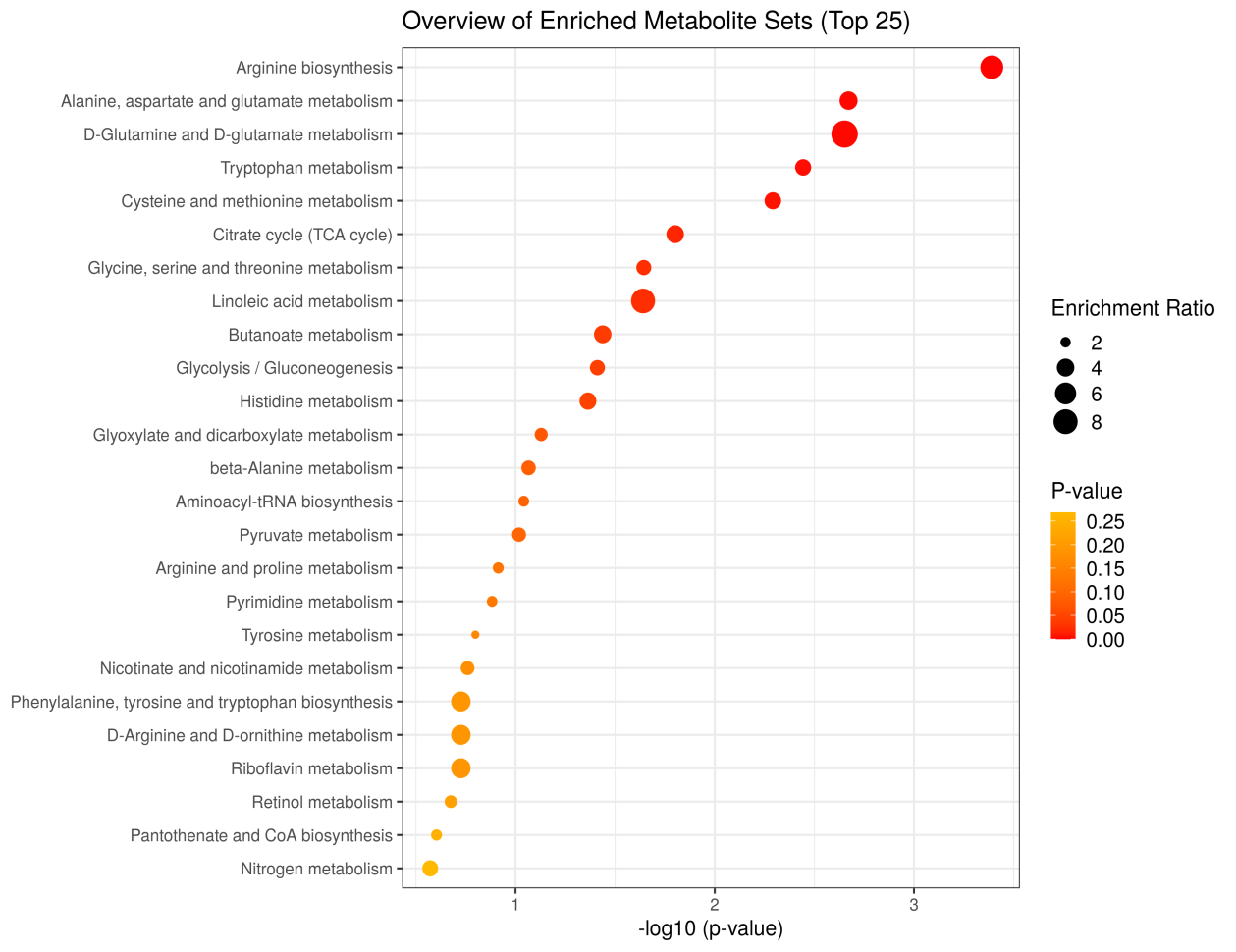


**A**

**B**

**Supplementary Figure S2.** Results of pathway analysis. **(A)** Bubble chart of pathway analysis. **(B)** Enrichment analysis.


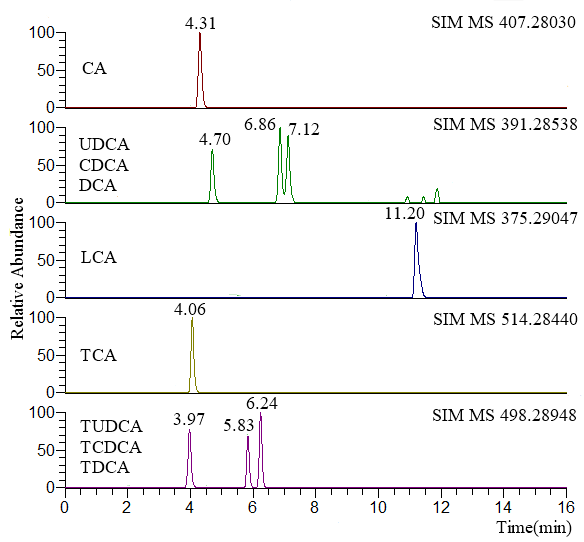

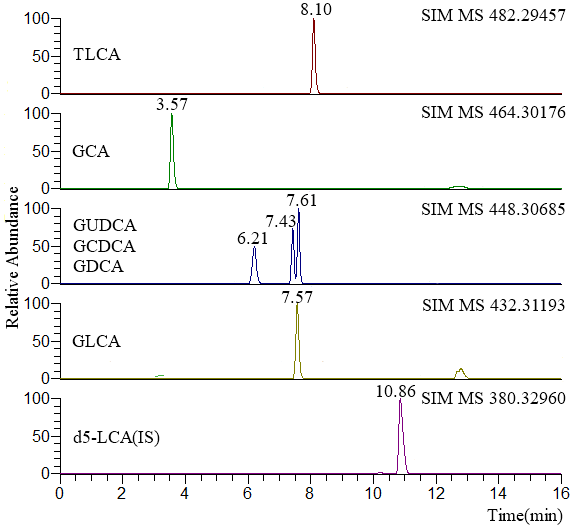


**Supplementary Figure S3.** Chromatographic separation of 15 bile acids and internal standard.


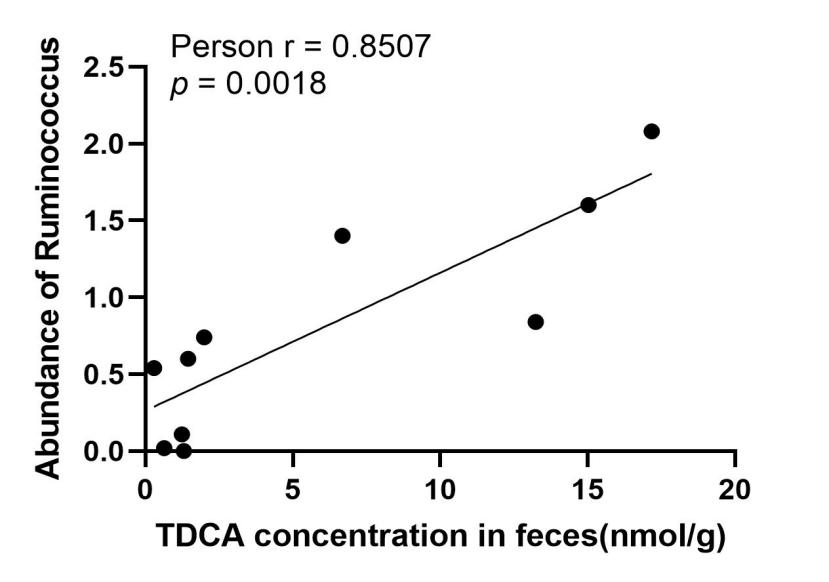

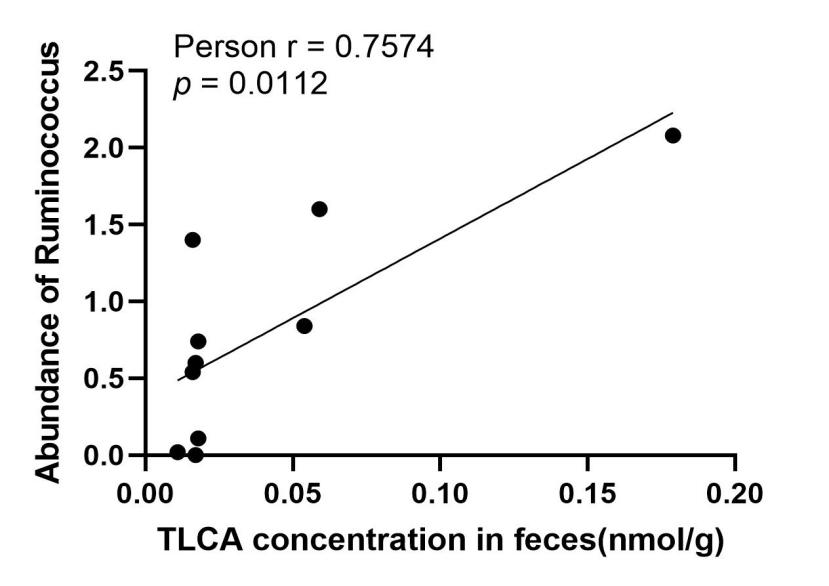

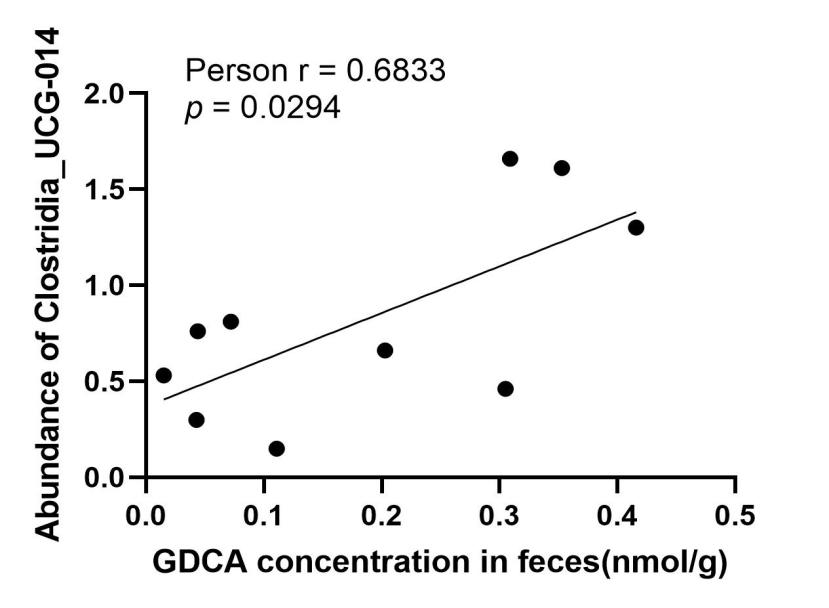

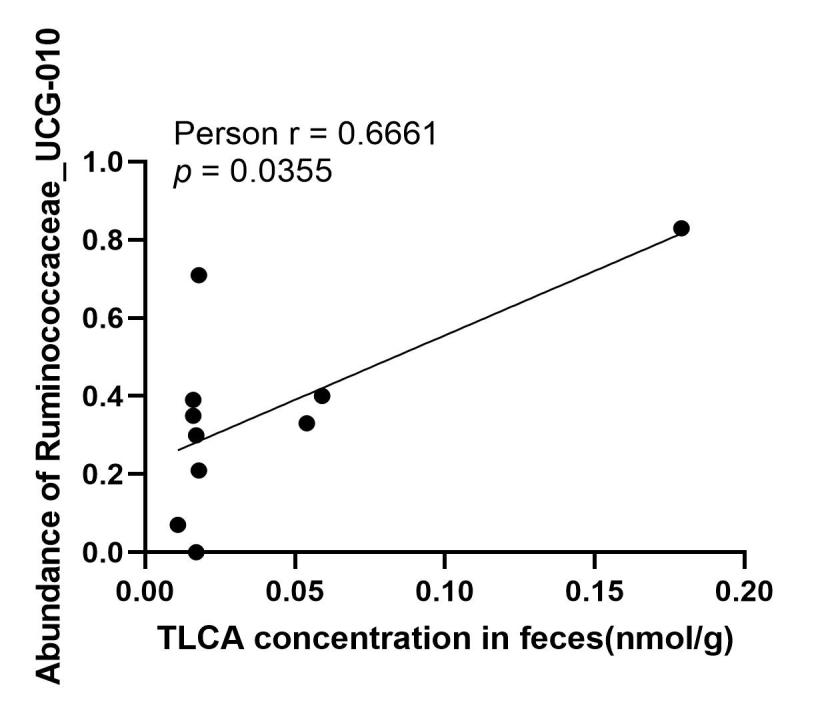


A

B

C

D

**Supplementary Figure S4.** Pearson’s correlation and linear regression analysis of the identified microbiotic abundance and secondary bile acid concentrations in the feces.

**Supplementary Table S1.**The proportion of solvent during gradient elution for untargeted metabonomics.FA:formic acid, AF:ammonium formate, ACN:acetonitrile

| Time(min) | solvent A(%) | solvent B(%) |
| --- | --- | --- |
|  | pos:0.1%FA-water  neg:5mM AF-water | pos:0.1%FA-ACN  neg:ACN |
| 0 | 98 | 2 |
| 1 | 98 | 2 |
| 9 | 50 | 50 |
| 12 | 2 | 98 |
| 13.5 | 2 | 98 |
| 14 | 98 | 2 |
| 20-pos/17-neg | 98 | 2 |

**Supplementary Table S2.** Detail KEGG pathway analysis results

| Pathway | Total | Hits | *p* value | FDR | Impact |
| --- | --- | --- | --- | --- | --- |
| Arginine biosynthesis | 14 | 5 | 0.0004 | 0.0359 | 0.1929 |
| Alanine, aspartate and glutamate metabolism | 28 | 6 | 0.0022 | 0.0944 | 0.5577 |
| Tryptophan metabolism | 41 | 7 | 0.0038 | 0.1070 | 0.3269 |
| Cysteine and methionine metabolism | 33 | 6 | 0.0054 | 0.1130 | 0.1292 |
| Histidine metabolism | 16 | 4 | 0.0072 | 0.1208 | 0.1393 |
| Citrate cycle (TCA cycle) | 20 | 4 | 0.0164 | 0.2293 | 0.1847 |
| beta-Alanine metabolism | 21 | 4 | 0.0195 | 0.2336 | 0.1605 |
| Linoleic acid metabolism | 5 | 2 | 0.0233 | 0.2451 | 1.0000 |
| Glycine, serine and threonine metabolism | 34 | 5 | 0.0266 | 0.2482 | 0.2556 |
| D-Glutamine and D-glutamate metabolism | 6 | 2 | 0.0339 | 0.2805 | 0.5000 |
| Butanoate metabolism | 15 | 3 | 0.0375 | 0.2805 | 0.0000 |
| Glycolysis / Gluconeogenesis | 26 | 4 | 0.0401 | 0.2805 | 0.2078 |
| Tyrosine metabolism | 42 | 5 | 0.0592 | 0.3826 | 0.1151 |
| Glyoxylate and dicarboxylate metabolism | 32 | 4 | 0.0766 | 0.4596 | 0.0661 |
| Aminoacyl-tRNA biosynthesis | 48 | 5 | 0.0941 | 0.5167 | 0.1667 |
| Pyruvate metabolism | 22 | 3 | 0.0984 | 0.5167 | 0.2068 |
| Arginine and proline metabolism | 38 | 4 | 0.1253 | 0.6190 | 0.2217 |
| Pyrimidine metabolism | 39 | 4 | 0.1344 | 0.6274 | 0.0957 |
| Nicotinate and nicotinamide metabolism | 15 | 2 | 0.1765 | 0.7136 | 0.0316 |
| Phenylalanine, tyrosine and tryptophan biosynthesis | 4 | 1 | 0.1895 | 0.7136 | 0.0000 |
| D-Arginine and D-ornithine metabolism | 4 | 1 | 0.1895 | 0.7136 | 0.0000 |
| Riboflavin metabolism | 4 | 1 | 0.1895 | 0.7136 | 0.5000 |
| Retinol metabolism | 16 | 2 | 0.1954 | 0.7136 | 0.4790 |
| Fructose and mannose metabolism | 18 | 2 | 0.2338 | 0.8183 | 0.2736 |
| Pantothenate and CoA biosynthesis | 19 | 2 | 0.2532 | 0.8507 | 0.0286 |
| Nitrogen metabolism | 6 | 1 | 0.2705 | 0.8660 | 0.0000 |
| Arachidonic acid metabolism | 36 | 3 | 0.2784 | 0.8660 | 0.3192 |
| Pentose phosphate pathway | 22 | 2 | 0.3114 | 0.9342 | 0.1356 |
| Valine, leucine and isoleucine biosynthesis | 8 | 1 | 0.3435 | 0.9619 | 0.0000 |
| Taurine and hypotaurine metabolism | 8 | 1 | 0.3435 | 0.9619 | 0.0000 |
| Ubiquinone and other terpenoid-quinone biosynthesis | 9 | 1 | 0.3773 | 1.0000 | 1.0000 |
| Ascorbate and aldarate metabolism | 10 | 1 | 0.4093 | 1.0000 | 0.0000 |
| Glutathione metabolism | 28 | 2 | 0.4242 | 1.0000 | 0.0268 |
| Phenylalanine metabolism | 12 | 1 | 0.4686 | 1.0000 | 0.2381 |
| alpha-Linolenic acid metabolism | 13 | 1 | 0.4960 | 1.0000 | 0.0000 |
| Starch and sucrose metabolism | 15 | 1 | 0.5466 | 1.0000 | 0.0068 |
| Sphingolipid metabolism | 21 | 1 | 0.6704 | 1.0000 | 0.0000 |
| Primary bile acid biosynthesis | 46 | 2 | 0.6935 | 1.0000 | 0.0229 |
| Propanoate metabolism | 23 | 1 | 0.7037 | 1.0000 | 0.0406 |
| Lysine degradation | 25 | 1 | 0.7336 | 1.0000 | 0.0470 |
| Galactose metabolism | 27 | 1 | 0.7606 | 1.0000 | 0.0000 |
| Porphyrin and chlorophyll metabolism | 30 | 1 | 0.7961 | 1.0000 | 0.0000 |
| Biosynthesis of unsaturated fatty acids | 36 | 1 | 0.8523 | 1.0000 | 0.0000 |
| Amino sugar and nucleotide sugar metabolism | 37 | 1 | 0.8600 | 1.0000 | 0.0873 |
| Purine metabolism | 66 | 1 | 0.9711 | 1.0000 | 0.0046 |
